# Severe Malaria Associated with* Plasmodium falciparum* and* P. vivax* among Children in Pawe Hospital, Northwest Ethiopia

**DOI:** 10.1155/2016/1240962

**Published:** 2016-03-07

**Authors:** Getachew Geleta, Tsige Ketema

**Affiliations:** Department of Biology, College of Natural Sciences, Jimma University, P.O. Box 378, Jimma, Ethiopia

## Abstract

Despite rigorous effort made to control malaria for more than a century, it is still among the main public health problems in least developed regions of the world. Majority of deaths associated with malaria occur in sub-Sahara Africa among biologically risked groups. Thus, this study was designed to assess the incidence of severe malaria syndromes among children in Pawe Hospital, Northwest Ethiopia. Children seeking medication for malaria infection in Pawe Hospital during the study period were recruited. Sociodemographic characteristics, physical, hematological, and clinical features of complicated malaria were assessed following standard parasitological and clinical procedures. A total of 263 children were found malaria positive. Among these, 200 were infected with* Plasmodium falciparum*. Most of the severe malaria symptoms were observed among children infected with* P. falciparum* and* P. vivax*. The study showed that significant number of the children developed severe life threatening malaria complications. This calls for prompt early diagnosis and effective treatment of patients to reduce mortality and complications associated with malaria in the study site.

## 1. Introduction

Malaria is one of the life threatening infections caused by protozoan parasite. It is still a major public health concern of most endemic areas of the world. Five human* Plasmodium* species (*Plasmodium falciparum, P. vivax, P. ovale, P. knowlesi,* and* P. malariae*) [1] cause malaria infection. The major complications are caused by* P. falciparum* and* P. vivax*, with* P. falciparum* being the more virulent. It is indicated that about 1–3 million mortalities per year, mainly in children and pregnant women, are due to severe malaria caused by* P. falciparum* [2]. These pathologies are severe anemia, cerebral malaria, and acute respiratory distress [3]. According to WHO/UNICEF report [4], of all malaria cases in the world, 60% were occurring in Africa. Of the 75% of global* P. falciparum* malaria cases, 80% mortality is documented in the same region.

Although the public health importance of* P. vivax* is overshadowed by* P. falciparum*, it is the most important parasite in Asia and South America. It accounts for about 390 million clinical cases annually [5]. Apart from these, studies from Indonesia, Papua New Guinea, Latine America Guyana, India, and Ethiopia show a strong association of this parasite with severe malaria symptoms [6–11].

According to President's Malaria Initiative [12] of Ethiopia, malaria is ranked as the leading communicable disease in Ethiopia, accounting for about 30% of the overall disability adjusted life years lost. Approximately 75% of the country is malarious with ~68% of the total population living in areas at risk of malaria. As reported by Ethiopia's Federal Ministry of Health (FMOH) [13], in 2009, malaria was the first cause of outpatient visits, health facility admissions, and inpatient deaths, accounting for 12% of outpatient visits and 9.9% of admissions. Thus, this study was designed to assess incidence of severe malaria syndromes associated with* P. falciparum* and* P. vivax* among children in one of the malaria endemic areas in Ethiopia.

## 2. Materials and Methods

### 2.1. Study Area

**Figure 1:**
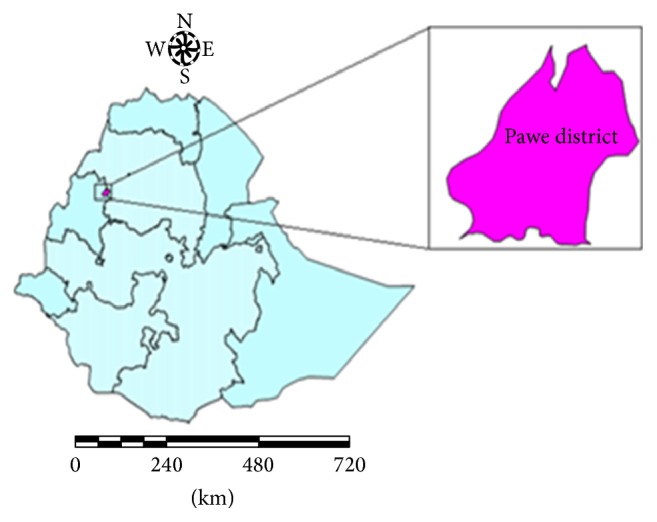
Map of the study site.

A descriptive hospital based study was conducted at Pawe district, Benishangul Gumuz Regional State, Northwest Ethiopia (Figure 1). The study site is geographically located at 11.009′ N latitude, 36.003′ E longitude, and an altitude of 1050 meters above sea level (masl). In this district, the major and minor malaria transmission peak seasons are from September to December and from April to May, respectively, coinciding with the major harvesting and planting seasons. The peak rainfall occurs from July to August. The mean annual rain fall and maximum temperature of the area are 1555.1 mm and 32°C (with mean monthly values ranging in 27–37°C), respectively. Pawe district has a total population of 45,552, of whom 23,265 were men and 22,287 were women. About 10,068 (22.1%) of population were urban inhabitants [14]. In the hospital, patients due to* P. falciparum* and* P. vivax* malaria are treated with Coartem (artemether-lumefantrine, 20 mg base/kg) and chloroquine (25 mg base/kg), respectively, following National Diagnosis and Treatment Guideline [15]. Moreover, as the hospital has all facilities for malaria diagnosis standard microscopic investigation was carried out for all patients following standard procedure recommended by WHO [16].

### 2.2. Study Population and Sample Size

Malaria infected children seeking medication at Pawe Hospital and fulfilling the inclusion criteria were considered in the study. Accordingly, children < 10 years, symptomatic, malaria positive, with no prior medication to the current illness, willing to participate in the study, and without chronic infections (none admitted to ART and Tb clinics) were enrolled in the study.

### 2.3. Data Collection

All guardians of children were provided with informed consent, and the sociodemographic and clinical data (age, weight, height, bed net utilization, fever, headache, presence of vomiting and diarrhea, prior medication, and body temperature) and any history of fever of the children were collected using predesigned data record form by health professionals working in the hospital. Also, signs of complicated malaria symptoms such as prostration, jaundice, impaired consciousness, hyperpyrexia, hyperparasitemia, persistent vomiting, respiratory distress, and hemoglobinuria were further assessed for all participants by trained physician and other health professionals at the hospital in accordance with WHO guidelines for the treatment of malaria [16].

### 2.4. Parasitological and Hematological Tests

About 2 mL of blood samples from each participant was collected in EDTA precoated tubes. Few drops were used for parasite identification and count. The remaining blood sample was used for further analysis. Briefly, two drops of the blood sample was collected on clean glass slide for preparation of thin and thick blood smears in duplicate. Thick and thin blood smears were stained with 10% Giemsa (pH = 7.2, for 10 min), while thin smears were fixed in methanol prior to Giemsa staining. Malaria parasites were identified under a microscope (100x oil immersion field) and parasite load was calculated after counting asexual parasites per 200 white blood cells (WBC) using the following formula [16], assuming that the mean WBC count of human is 8,000/*μ*L:

(1) $$\begin{array}{c} \text{Parasite count}/\mu L=\frac{\text{Number of observed asexual parasites}\times 8000\text{ WBC count}/\mu L}{200\;\;WBCs}. \end{array}$$

The remaining blood sample was used for measurement of hematological parameters such as hemoglobin (Hb), hematocrit (HCT), neutrophils, eosinophils, monocytes, and lymphocytes using autosampler CBC machine (Automated CBC Analyzer: Sysmex KX-21).

Severe malaria symptoms were classified following WHO guideline for the management of severe malaria as severe anemia (Hb < 5 g/dL or hematocrit level < 15%), hyperparasitemia (parasite load > 100,000 parasites/*μ*L), persistent vomiting, respiratory distress, convulsion (more than two in 24 hours), comma, hemoglobinuria (discoloration of urine), prostration [unable to walk, sit, and stand or unable to feed and drink in infants], and hyperpyrexia (body temperature > 40°C) [17–19].

### 2.5. Data Analysis

Data were analyzed using SPSS software (version 20.0, Armonk, NY: IBM Corp). Descriptive statistical tests were used for analysis of clinical, demographic, and parasitological data. Association between variables was evaluated using Pearson correlation test. Median (range) was considered over mean (SD) for nonnormally distributed variables. Multiple logistic-regression model was used to assess predictive variables of severe malaria complications. One-way ANOVA (analysis of variance) was employed to compare WBC indices. In all analysis, significance level was considered at 95% confidence interval (*P* < 0.05).

### 2.6. Ethical Consideration

The study was ethically approved by Research and Ethical Review Committee of College of Natural Sciences, Jimma University, Ethiopia. From all study participants, oral and written assent were obtained prior to data collection. Malaria positive cases were treated according to national malaria diagnosis and treatment guidelines [15].

## 3. Results

### 3.1. Prevalence of Malaria in the Study Area

**Figure 2:**
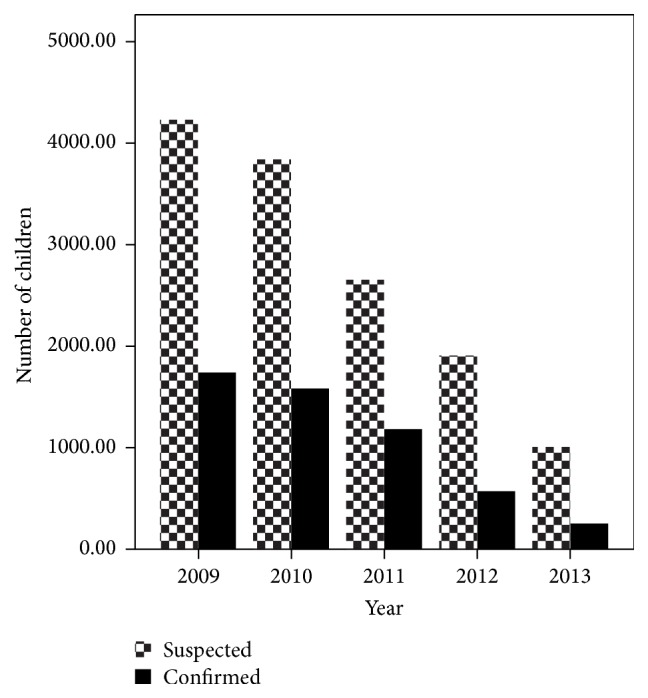
Five-year malaria prevalence report among children in Pawe Hospital, Northwest Ethiopia (Source: Annual reports of Pawe Hospital, unpublished data).

Malaria was still among the top health concerns in the study area. Although the trend of malaria positive cases is showing an overall declining pattern, the five-year malaria prevalence data from the hospital (2009–2013) revealed that the numbers of infected children ≤ 10 years are still higher (Figure 2). The prevalence of malaria among children over the five-year period in the hospital was reduced from 41.45% (2009) to 25.7% (2013).

### 3.2. Sociodemographic and Clinical Characteristics of the Study Participants

**Figure 3:**
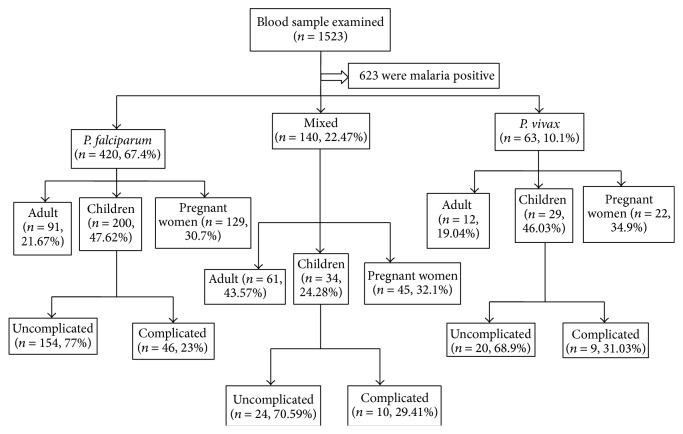
Flow chart of the study.

During the study period, a total of 1523 blood samples were collected from presumptive malaria patients (Figure 3). Among these, 263 (17.27%) were children, of which 139 (52.85%) and 124 (47.14%) were males and females, respectively.

The median age of children enrolled in this study was 4.1 years (one month–10 years). Mean auxiliary body temperature of the children was 38.14°C. Proportion of children with vomiting and diarrhea symptoms was 162 (61.59%) and 94 (35.74%), respectively. Bed net coverage was very limited, with only 76 (28.89%) of the study participants using bed net. Mean hemoglobin and HCT level were 7.78 g/dL and 27.52%, respectively. Geometric mean parasite count was 9898 parasites/*μ*L (Table 1). The number of anemic children (Hb level < 11 g/dL) encountered was 194 (73.76%).

### 3.3. Clinical Characteristics of Children Infected with Different* Plasmodium* spp

Geometric mean parasite count (asexual stage) and mean body temperature for* P. falciparum* and* P. vivax* infected children were 10963 and 3902 parasites/*μ*L and 38.63 and 38.62°C, respectively. Some participants had symptom of splenomegaly due to* P. falciparum* (*n* = 6, 60%) and* P. vivax* (*n* = 2, 20%). About 15 (12.4%) of the study participants were self-reported for self-medication prior to coming to the health facility. The BMI of the study participants were 18.44, 19.15, and 19.30 for mixed, mono-*P. falciparum,* and* P. vivax* infection, respectively (Table 2).

Although there was no significant association (*r* = 0.038, *P* = 0.54) between age and Hb level, as age of children increased Hb level also showed an increment. Most of the children with hemoglobin level <5 g/dL (an indicator of severe anemia) were found in age group <1 year.

There were no significant differences (*P* > 0.05) in level of WBC indices among children infected with different* Plasmodium* species. In* P. falciparum* and mixed infected children, counts of neutrophils, eosinophil, and monocytes had similar pattern, except for lymphocyte whose level rose in* P. falciparum* infection. However, in* P. vivax* infected children eosinophils and lymphocytes were lower, but other WBC indices such as monocytes and neutrophils were higher (Table 3).

#### 3.3.1. Incidence of Severe Malaria Symptoms among Children Infected with Different* Plasmodium* spp

Among the 263 study participants enrolled in the study, a total of 46* P. falciparum* positive children showed at least one of the WHO recommended criteria of severe malaria. These were severe anemia, 36 (18%), prostration, 41 (24%), hyperpyrexia, 16 (8%), respiratory distress, 10 (5%), hemoglobinuria, 14 (7%), persistent vomiting, 16 (8%), and hyperparasitemia, 33 (16.5%), but none had pathology of hepatomegaly and comma. These symptoms of severe malaria complications among the children were significantly higher (*P* < 0.05) than other* Plasmodium* infections except symptoms such as confusion and splenomegaly (Table 4). Likewise, a total of 29 of children enrolled in the study were infected with* P. vivax* malaria. Among these cases, those that showed symptoms of severe malaria complications and the respective symptoms were 4 (13.8%), severe anemia, 9 (31.03%), prostration, 7 (24.13%), hyperpyrexia, 3 (10.3%), respiratory distress, 2 (6.89%), hemoglobinuria, 1 (3.4%), confusion, and 1 (3.4%), persistent vomiting, but none had symptoms such as hyperparasitemia, hepatomegaly, and comma (Table 4).

Logistic-regression analysis identified that there were three key predictive indicators of severe malaria: respiratory distress, hyperpyrexia, and severe anemia [odd ratio (OR) = 1.26, 95% CI = 1.115–2.59, OR = 1.033, 95% CI = 1.04–2.01, and OR = 1.325, 95% CI = 1.56–3.14, resp.]. These severe malaria symptoms were highly prevalent among children less than five years. But other severe malaria symptoms were not significantly different between children of age groups < 5 and > 5 years infected with* P. falciparum*.

## 4. Discussion

Although the prevalence of malaria has been showing declining pattern in some parts of Ethiopia [20], in the current study area it is still the top health concern. The number of infected children aged ≤ 10 years was still higher. According to the five-year prevalence report of the hospital, trend of malaria positive cases showed reduction. This overall decreasing pattern was similar to earlier report made on time series analysis of trends in malaria cases from Ethiopia [20]. The decline pattern was accounted for intensive malaria intervention strategies that involved vector control and symptomatic cases treatment as a result of which the morbidity and mortality associated with malaria decreased [20, 21]. According to Aregawi et al. [20], the status of inpatients and malaria related death was reduced to 55% and 85%, respectively, during 2001–2011 in the same hospital.

Although the two* Plasmodium* species,* P. falciparum* and* P. vivax*, are important parasites in malaria related problems in Ethiopia,* P. falciparum* was the most predominant species accounting for about 76% of all malaria infections among children assessed in this study.

Malaria is a deadly disease to all humanity. But the most vulnerable (biologically at the highest risk) are infants and young children (due to their underdeveloped immunity) and pregnant women (as their immunity reduces during malaria infection). In pregnant women it causes increased risk of abortion, stillbirth, premature delivery, and low-birth weight infants besides mortality [22, 23]. Thus, severe malaria cases documented among children in the current study evidence the higher risk of these groups to malaria associated morbidity. Furthermore, incidence of severe malaria associated with* P. vivax* strengthens the fact that this parasite is no more mild; rather it could be responsible for some life threatening complications among children in malaria endemic regions [6, 9, 24–26].

The observed severe malaria complications were much lower than the earlier reports made from Papua New Guinean, Ghana, and Yemen [27–29]. This variation of manifestation of severe malaria in these regions could be due to differences in intensity of malaria transmission. In areas where there is low or moderate transmission, the incidence of severe malaria appears the highest [30].

Most of the children enrolled in this study were found in age group ≤5 years. In this age category higher load of parasitemia, respiratory distress, and incidence of severe anemia were observed. This might be due to the development of poor immunity against the disease [31] although they could gradually develop protective immunity to malaria as they get older and repeatedly exposed to the disease [32]. This could be supported by the observed reduction of anemic condition with age possibly due to elimination of the parasite by the immunity and reducing the hemolysis of RBCs.

Usually anemia is a hallmark of* P. falciparum* infection due to intense hemolysis (destruction) of infected RBCs due to higher parasitemia caused by the parasite. Unlike other* Plasmodium* species,* P. falciparum* infect all types of RBCs found at different stages of development (from immature young to old RBCs). With additional hemolysis of noninfected RBCs by host immunity, the likely occurrence of severe anemia in* P. falciparum* infected children is high. However, due to selective preference to only young RBCs by* P. vivax*, it appears that the number of haemolysed RBCs during* P. vivax* infection is minimal. Thus, the incidence of severe anemia associated with* P. vivax* might occur as a result of rigor inflammatory reactions due to proinflammatory response and cytokines activation [33] and less deformability of RBCs during* P. vivax* infection [34, 35]. On the other hand, the rate of noninfected RBCs hemolysis for every infected RBC destroyed could contribute to the incidence of anemia as number of nonparasitized RBCs removed from circulation during* P. vivax* is much higher (~32) than* P. falciparum* (~8) [36]. Furthermore, lack of significant difference in levels of Hb and HCT between* P. falciparum* and* P. vivax* positive children strengthens the fact that* P. vivax* was one of the risk factors associated with incidence of severe anemia among children.

Although bed net is unquestionable tool for prevention of malaria vector and widely used in most malaria endemic areas of the country, in the current study its distribution and utilization among the biologically risked groups, supposed to get priority in the family, were very limited and only few were sleeping under bed net. This situation might be among the most important factors aggravating the highest prevalence of malaria in the study area, when the current trend of malaria infection dramatically dropped in most malaria endemic areas of the country.

## 5. Conclusion

Severe malaria symptoms associated with* P. falciparum* were observed in significant number of children assessed in this study.* P*.* vivax* caused severe malaria complications documented in the current study strengthen the fact that this parasite is no longer benign. Thus, early detection of infected cases and implementation of effective treatment should be in practice to reduce mortality and morbidity associated with malaria in the current study area.

## Figures and Tables

**Table 1:** Sociodemographic and clinical manifestation of uncomplicated malaria infected children in Pawe Hospital, Northwest Ethiopia.

Characteristics	Proportion
Age: median (range)	4.1 (1 month–10 years)
Sex:	
Male	139 (52.8%)
Female	124 (47.14%)
Mean body temperature (°C)	38.14 ± 0.21
Bed net utilization	76 (28.89%)
BMI (Body Mass Index) (kg/m^2^)	19.15 ± 0.58
Vomiting	162 (61.59%)
Diarrhea	94 (35.74%)
Mean Hb level (g/dL)	7.78 ± 2.4
Mean hematocrit level (HCT) (%)	27.52 ± 4.91
Geometric mean parasite (parasite/*µ*L)	9898 ± 5138.2
Headache	141 (53.6%)
Premedication	92 (34.98%)
Anemia (mild)	194 (73.76%)

**Table 2:** Clinical features of malaria infected children with respect to different spp. of *Plasmodium* infections in Pawe Hospital, Northwest Ethiopia.

Characteristics	*P. f* (*n* = 200)	Mix (*n* = 34)	*P. v* (*n* = 29)
Temperature (°C)	38.63 ± 0.25	38.62 ± 0.73	38.62 ± 0.14
Bed net	42 (55.26)	23 (30.26)	11 (14.47)
Hb (g/dL)	7.87 ± 1.03	8 ± 3.6	8 ± 2.69
HCT (hematocrit) (%)	27.52 ± 2.4	28.14 ± 1.98	26.17 ± 3.11
Mean parasite (parasite/*µ*L)	10963 ± 3692	7923 ± 2756	3902 ± 1569
BMI (kg/m^2^)	19.15 ± 1.28	18.44 ± 0.99	19.2 ± 1.1
Vomiting	119 (73.45)	27 (16.67)	16 (9.87)
Diarrhea	68 (72.34)	18 (19.15)	8 (8.5)
Hepatomegaly	0 (0)	0 (0)	0 (0)
Splenomegaly	6 (60)	2 (20)	2 (20)

*P. f* = *P. falciparum*, *P. v* = *P. vivax*, Mix = mixed infection; numbers within bracket are %.

**Table 3:** Levels of WBC indices among children infected with different *Plasmodium* species at Pawe Hospital, Northwest Ethiopia.

WBC indices	Mean ± SD			*P* value
	*P. f* (*n* = 200)	*P. v* (*n* = 29)	Mix (*n* = 34)	
Neutrophil	46.4 ± 15.1	47.82 ± 13.06	46.7 ± 23.13	0.816
Lymphocyte	29.03 ± 10.9	27 ± 12.63	28.2 ± 20.55	0.701
Eosinophil	14.9 ± 9.76	13.3 ± 11.99	14.7 ± 11.68	0.769
Monocyte	9.12 ± 8.56	11.4 ± 10.36	8.85 ± 13.02	0.252

*P. f* = *P. falciparum*, *P. v* = *P. vivax*, Mix = mixed infection.

**Table 4:** Incidence of severe malaria symptoms among *P. falciparum* and *P. vivax* monoinfected children, Pawe Hospital, Northwest Ethiopia.

Characteristics	Proportion (%)	
	*P. falciparum* (*n* = 200)	*P. vivax* (*n* = 29)
Severe anemia (Hb < 5 g/dL or HCT < 15%)	36 (18)	4 (13.8)
Hyperparasitemia (>100,000 parasite/*µ*L)	33 (16.5)	0 (0)
Prostration	41 (24)	9 (31.03)
Hyperpyrexia (≥40°C)	40 (20)	7 (24.13)
Confusion	3 (1.5)	1 (3.4)
Splenomegaly	6 (3)	2 (6.89)
Respiratory distress	10 (5)	3 (10.3)
Hemoglobinuria	14 (7)	2 (6.89)
Persistent vomiting	16 (8)	1 (3.4)
Comma	0 (0)	0 (0)
